# Asymmetric Dimethylarginine Plasma Levels and Endothelial Function in Newly Diagnosed Type 2 Diabetic Patients

**DOI:** 10.3390/ijms131113804

**Published:** 2012-10-24

**Authors:** Angela Sciacqua, Nadia Grillo, Michele Quero, Giorgio Sesti, Francesco Perticone

**Affiliations:** Department of Medical and Surgical Sciences, University Magna Græcia of Catanzaro, Campus Germaneto, viale Europa, 88100, Italy; E-Mails: sciacqua@unicz.it (A.S.); nadia_grillo@libero.it (N.G.); michelequero@yahoo.it (M.Q.); sesti@unicz.it (G.S.)

**Keywords:** endothelium, type 2 diabetes, asymmetric dimethylarginine

## Abstract

It is now well established that major risk factors for cardiovascular diseases (CVD) impact upon endothelial function by decreasing nitric oxide (NO) bioavailability. Asymmetric dimethylarginine (ADMA), an endogenous analog of l-arginine, is able to inhibit the activity of endothelial-NO synthase, promoting endothelial dysfunction. Type 2 diabetes (T2D) is characterized by a reduced endothelium-dependent vasodilation and increased ADMA levels and ADMA is strongly associated with micro- and macrovascular diabetic complications. However, there are not a lot of data about the role of ADMA on endothelial function in newly diagnosed T2D patients without cardiovascular (CV) complications. For this aim, we have enrolled forty-five newly diagnosed T2D patients, evaluated by a oral glucose tolerance test, and thirty normal subjects. Endothelium-dependent and -independent vasodilatation was investigated by intra-arterial infusion of increasing doses of acetylcholine (ACh) and sodium nitroprusside. ADMA was measured by high-performance liquid chromatography and insulin resistance (IR) by HOMA. Newly diagnosed T2D patients showed higher ADMA and l-arginine mean values in comparison with normal subjects and a significantly reduced ACh-stimulated forearm blood flow (FBF). In T2D patients FBF was significantly and inversely correlated with ADMA (*r* = −0.524, *p* < 0.0001) and in a multivariate regression analysis, ADMA resulted the stronger predictor of FBF, explaining the 27.5% of variability (*p* < 0.0001). In conclusion, ADMA was strongly related to endothelial dysfunction also in patients with newly diagnosed T2D, without clinically manifest vascular complications. This field is of great interest for understanding the mechanisms underlying the pathogenesis of diabetic disease and its CV complications.

## 1. Introduction

The normal endothelium, with its anatomical and functional integrity, plays a pivotal role in the prevention of the both coronary and extracoronary atherosclerosis [1,2]. All vascular protective effects of the endothelium, as well as inhibition of monocyte and leukocyte adhesion and platelet aggregation, modulation of vascular smooth muscle cells and fibroblasts proliferation [2,3], are regulated by nitric oxide (NO), a short-lived molecule produced by the endothelial nitric oxide synthase enzyme (e-NOS) from the amino acid l-arginine [4].

It is well known that all traditional risk factors for cardiovascular diseases (CVD), such as hypertension and diabetes, [5,6] affect endothelial function by decreasing NO bioavailability. This condition known as “endothelial dysfunction” may be caused by several mechanisms including decreased NO synthesis, increased NO degradation due to oxidative stress, or reduced sensitivity of smooth muscle cell to NO [2,3,7,8]. Thus, endothelial dysfunction represents an early step in the atherosclerotic process and an independent predictor of cardiovascular events [9]. With regard to the first mechanism, the activity of e-NOS may be inhibited by endogenous analogs of l-arginine, such as asymmetric dimethylarginine (ADMA) [10], that derive from the catabolism of proteins containing methylated arginine residues. In keeping with this, there are several evidences that ADMA is increased in different clinical settings; as with chronic renal diseases [11], hypercholesterolemia [12], essential hypertension [13] and diabetes mellitus [14,15], all conditions are associated with the appearance and progression of atherosclerotic disease.

Interestingly, several recent findings highlight the importance of the prediabetic condition and the pathogenetic mechanism of insulin-resistance (IR) in the appearance and progression of subclinical organ damage [16–18]. In keeping with this, we also demonstrated that normotolerant subjects with 1-hour postload plasma glucose >155 mg/dL have a worse metabolic and vascular profile in comparison with normotolerant subjects with 1-hour postload plasma glucose <155 mg/dL [19–21]. Of clinical relevance, all these modifications are attributable to different degrees of IR observed between groups [22]. In addition, IR has a main role in promoting vascular damage; in fact, endothelial dysfunction can be observed early in the spectrum of IR, before the detection of clinical hyperglycemia [23,24]. In physiological conditions, insulin is a vasodilating substance, able to stimulate e-NOS expression and, thus, endothelial NO production. The phosphatidylinositol 3-kinase (PI-3K) pathway is critical for insulin-mediated glucose uptake into target tissues, regulating insulin-dependent endothelial NO production [25]. In conditions of IR, a systemic defect in the PI-3K pathway leads to a combined defect in insulin-mediated glucose transport and in insulin-stimulated endothelial vasodilation [26,27], favoring the proatherogenic effect of insulin.

In addition to these mechanisms, ADMA may have a crucial role in promoting vascular damage in T2D and the close correlation between ADMA levels and IR may, at least in part, explain it. According with this, ADMA concentration increases in insulin-resistant subjects, and pharmacological treatment that improves IR is able to reduce ADMA levels [28]. Moreover, even if demonstrated in hypertensive nondiabetic patients, ADMA is a strong determinant of IR, and the coexistence of these two conditions acts in a multiplicative manner in promoting endothelial dysfunction [29].

However, there are not a lot of data about the role of ADMA on endothelial function in newly diagnosed T2D patients without cardiovascular (CV) complications. Thus, the aim of this study was to evaluate this association.

## 2. Results

**Figure 1:**
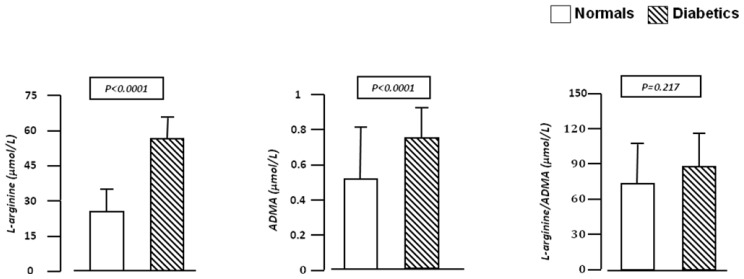
We graphically reported the plasma concentrations mean values of ADMA and l-arginine in normal subjects and newly diagnosed type 2 diabetic patients. ADMA and l-arginine mean values were significantly (*p* < 0.0001) higher in diabetic patients than in normal subjects, but there were no significant differences in mean l-arginine/ADMA ratio between groups.

Baseline demographic, hemodynamic, and humoral characteristics of the study population, according to normal and diabetic status, are summarized in Table 1. There were no significant differences in gender, age, BMI, waist circumference, BP, total and LDL-cholesterol and e-GFR between normal subjects and diabetic patients. In diabetic patients, fasting glucose (*p* < 0.0001), insulin (*p* < 0.0001), HOMA index (*p* < 0.0001), triglyceride (*p* = 0.004) and hs-CRP (*p* < 0.0001) mean values were significantly higher than in normal subjects. On the contrary, HDL-cholesterol mean values were significantly lower (*p* = 0.001). In addition, ADMA and l-arginine plasma concentrations were significantly (*p* < 0.0001) higher in diabetic patients than in normal subjects, but there were no significant differences in mean l-arginine/ ADMA ratio between groups (89.1 ± 27.6 *vs*. 78.5 ± 45.5; *p* = 0.217) (Figure 1).

### 2.1. Vascular Function

**Figure 2:**
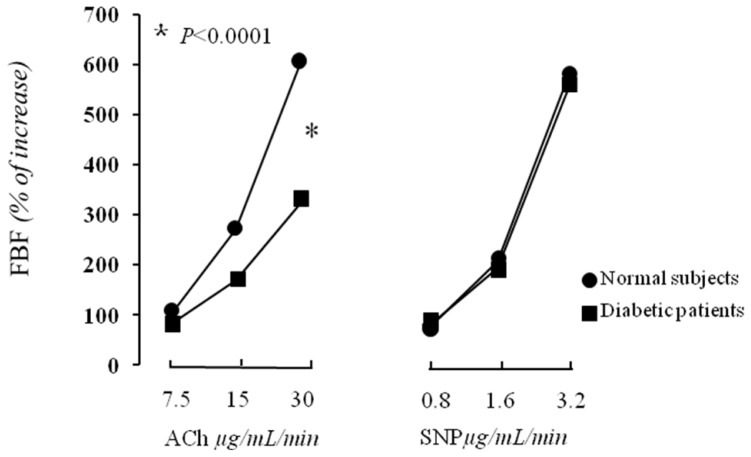
We graphically report forearm blood flow (FBF) increase during infusion of acetylcholine (Ach) (on the **left**), and sodium nitroprusside (SNP) (on the **right**). ACh-stimulated FBF was significantly reduced in newly diagnosed type-2 diabetic patients compared with normal subjects (*p < 0.0001). There was no significant difference in SNP-stimulated FBF between groups.

There was not any difference in baseline FBF between normal subjects and diabetics (3.2 ± 0.8 *vs*. 3.1 ± 0.3 mL·100 mL^−1^ of tissue min^−1^). Intra-arterial infusion of ACh induced a significant dose-dependent increase in FBF in both groups. In particular, the FBF values at the three incremental doses of ACh were 6.8 ± 3.4, 12.1 ± 4.7 and 22.5 ± 6.1 mL·100 mL^−1^ of tissue min^−1^ and 5.7 ± 2.1, 8.4 ± 3.3 and 13.2 ± 4.6 mL·100 mL^−1^ of tissue min^−1^ for normal subjects and newly diagnosed diabetic patients, respectively (Figure 2).

In addition, there was a significant decrease in forearm VR in both groups. The VR values at the three incremental doses of ACh were 17.1 ± 8.1, 9.2 ± 4.4 and 4.6 ± 1.1 U, and 18.8 ± 6.1, 13.2 ± 5.1 and 8.4 ± 3.4 U for normal subjects and diabetic patients, respectively. In consideration of this, newly diagnosed diabetic patients showed a reduced ACh-stimulated FBF in comparison with normal subjects (*p* < 0.0001). Incremental doses of intra-arterial infusion of SNP induced a significant increase in FBF as well as a decrease in forearm VR in both groups without significant difference between them (Figure 2). Intra-arterial infusion of ACH and SNP did not cause any significant change in BP or HR in both groups.

### 2.2. Correlational Analyses

As shown in Table 2, in diabetic patients the peak percent increase in ACh-stimulated FBF was significantly and inversely correlated with ADMA (*r* = −0.524, *p* < 0.0001), HOMA index (*r* = −0.428, *p* = 0.002), hs-CRP (*r* = −0.416, *p* = 0.002) and l-arginine (*r* = −0.261, *p* = 0.042). Conversely, ADMA was linearly correlated with HOMA (*r* = 0.342, *p* = 0.011), and hs-CRP (*r* = 0.348, *p* = 0.010). In normal subjects, only age was significantly associated with the peak increase in ACh-stimulated FBF (*r* = −0.320, *p* = 0.043).

Thus, in newly diagnosed type 2 diabetic patients, variables reaching statistical significance and gender, as dichotomic values, were inserted in a stepwise multivariate linear regression model to determine the independent determinants of the peak FBF response to ACh. As shown in Table 3, ADMA was the major determinant of FBF peak increase, explaining 27.5% of its variation (*p* < 0.0001), while HOMA index explains another 7% (*p* = 0.040) of its variation.

## 3. Discussion

The main finding of this study is that, newly diagnosed T2D patients, without clinically manifest vascular complications, had lower endothelium-dependent vasodilation and a worse metabolic and inflammatory profile in comparison with normal subjects. In addition, their ADMA and l-arginine plasma concentrations were significantly higher. Moreover, the endogenous inhibitor of e-NOS, ADMA, is inversely related to endothelial function and it represents the strongest determinant of ACh-stimulated FBF, accounting for a 27.5% of its variation. In addition, ADMA was strongly related with HOMA index and hs-CRP levels that participate in the atherosclerotic process.

Itis known that an impaired endothelium-dependent vasodilation can be observed early in diabetic blood vessels [6,30], and there are some evidences that endothelial dysfunction precedes the development of diabetes [31], confirming the coexistence of a bidirectional mechanism linking metabolic and vascular alterations. The clinical relevance of our data consists in the fact that all these alterations were also present in newly diagnosed diabetic patients with normal fasting plasma glucose.

Metabolic abnormalities associated with diabetes such as hyperglycemia, hyperinsulinemia/IR, and dyslipidemia together with oxidative stress may contribute to endothelial dysfunction, as well as ADMA levels, as demonstrated by several studies in both type 1 or 2 diabetes [6,30,32]. In accordance with this, the increase in ADMA plasma levels, in response to a high-fat meal, is significantly and inversely related to the decrease in endothelial function in T2D patients [33].

Several studies have demonstrated that ADMA, in T2D patients, is able to predict both micro- and macrovascular complications. In particular, Hanai and coworkers have showed, in an observational cohort study, that ADMA is a potent predictor of the progression of nephropathy in Japanese diabetic patients [34]. Moreover, Malecki and coworkers demonstrated a significant association between ADMA and retinopathy in T2D patients [35]. Similarly, Krzyzanowska and coworkers demonstrated the additive effect of ADMA and CRP in the prediction of cardiovascular events in T2D patients [36]. Our data consent to expanding these findings because they show that ADMA plasma levels are already increased in newly diagnosed T2D patients with normal fasting glucose. These evidences demonstrate that pathogenetic mechanisms, operating in vascular and metabolic alterations, are activated early, thereby amplifying the global cardiovascular risk profile also in subjects considered low risk.

Another relevant evidence provided by our study is that newly diagnosed T2D patients have higher levels of l-arginine in comparison with normal subjects, while the l-arginine/ADMA ratio was not significantly different. This finding is not surprising because this has been already observed in different settings of patients [13,32] and may be explained by the fact that also low circulating concentrations of ADMA are able to competitively inhibit eNOS at physiological concentrations resulting in a rightward shift of the concentration-response curve of l-arginine [37]. Moreover, in our study, ADMA plasma levels are associated with HOMA index confirming previously published data in different sets of patients [29,32]. This is relevant because insulin-mediated glucose uptake as well as the reactivity of vessels in insulin-sensitive tissues are modulated by NO generated in the endothelium; thus, IR represents an important pathogenetic mechanism in the development of endothelial dysfunction and T2D. Finally, our data confirm the strong association between ADMA and hs-CRP also in newly diagnosed T2D patients, suggesting that also mild vascular inflammation contribute to IR and endothelial dysfunction [31]. All these evidences confirm that some pathogenetic mechanisms are contemporary activated promoting reverberant circuits that, if not known and thus not treated early, may contribute to the appearance and progression of subclinical organ damage and CV outcomes.

## 4. Experimental Section

### 4.1. Study Population

The study included forty-five newly diagnosed type 2 diabetic patients, diagnosed by an oral glucose tolerance test (OGTT), and a control group of 30 normal subjects, who were recruited at the Department of Medical and Surgical Sciences, University Magna Graecia of Catanzaro.

All subjects were Caucasian and were participating in an observational prevention campaign for cardiometabolic risk factors, the CAtanzaro MEtabolic RIsk factors Study (CATAMERIS) as previously reported [38]. The groups were well matched for gender, age, body mass index (BMI), waist circumference and hemodynamic characteristics. After 12 h of fasting, individuals underwent anthropometric evaluation, and a venous blood sample was drawn for laboratory determinations. To avoid a possible interference between cigarette smoking and ADMA, we excluded from this study current or previous smokers. Readings of clinic blood pressure (BP) were obtained in the left arm of the supine patients after five min of quiet rest with a mercury sphygmomanometer. A minimum of three BP readings were taken on three separate occasions at least two weeks apart. Subjects were excluded if they had a history of arterial hypertension, other exclusionary criteria were chronic gastrointestinal diseases associated with malabsorption, chronic pancreatitis, history of any malignant disease, history of alcohol or drug abuse, liver or kidney failure, and treatments able to modify glucose metabolism. The 75 g OGTT was performed after 12-h fasting, using the World Health Organization (WHO) criteria. All control subjects were normoglucosetolerant during OGTT. Moreover, newly diagnosed T2D patients were included in the study if they did not present ketonuria or anti-GAD antibodies at the diagnosis. In addition, all patients had to present a normal renal function with creatinine value (<1.2 mg/dL in females and <1.4 mg/dL in males) and an absence of proteinuria. Creatinine measurements were carried out within days of the initial baseline examination by using Jaffe methodology and by the URICASE/POD (Boehringer Mannheim, Mannheim, Germany) method implemented in an auto-analyzer. Values of estimated glomerular filtration rate (e-GFR) (mL/min/1.73 m^2^) were calculated by using the new equation proposed by investigators in the chronic kidney disease epidemiology (CKD-EPI) collaboration. This equation was developed from a much large cohort of patients, including both normal and CKD individuals. We preferred this equation because it is more accurate in subjects with e-GFR > 60 mL/min/1.73 m^2^, as our patients were supposed to have considering the creatinine value <1.5 mg/dL [39]. Moreover, none of the patients had a history or clinical evidence of atherosclerotic complications, nor coronary artery disease or peripheral vascular disease. Finally, valvular heart disease, hyperlipidemia, coagulopathy, or any disease predisposed to vasculitis or Raynaud’s phenomenon were excluded. All participants were drug naïve.

The study was approved by the institutional ethics committee and informed written consent was obtained from each subject in accordance with the principles of the Declaration of Helsinki.

### 4.2. Laboratory Determinations

All laboratory measurements were performed after at least 12 fasting hours. Plasma glucose was determined immediately by the glucose oxidase method [Glucose analyzer, Beckman Coulter, Milan; intra-assay coefficient of variation (CV) 2.2%, inter-assay CV 3.8%]. Triglyceride, total, low and high-density lipoprotein (LDL, HDL) cholesterol concentrations were measured by enzymatic methods (Roche Diagnostics GmbH, Mannheim, Germany). Serum insulin was determined in duplicate by a highly specific radioimmunoassay using two monoclonal antibodies; intra-assay CV 2.1%, inter-assay CV 2.9%. Circulating IGF-1 was obtained in duplicate using a site-specific (crossreactivity: human insulin undetectable, intact proinsulin undetectable) and sensitive immunoradiometric assay (Nichols Advantage Kit from Nichols Institute Diagnostics, San Clemente, CA, USA; intra-assay CV 5.2%, inter-assay CV 5.7%; internal reference values in healthy subjects 71–360 ng/mL).

### 4.3. Determination of Insulin Resistance

Insulin sensitivity was estimated by using the previously validated homeostasis model assessment (HOMA) index, calculated from the fasting glucose and insulin concentrations according to the formula: HOMA=[insulin (μU/mL) × glucose (mmol/L)]/22.5. HOMA has been commonly used in clinical studies as well as in a population-based study and it is highly correlated with IR measured by euglycemic clamp [40].

### 4.4. Determination of ADMA and l-Arginine

This method has been previously described as our group [13,29]. Briefly, samples were stored in prechilled vacutainers containing edetic acid, placed immediately on ice, and centrifuged within 30 min at 4 °C; plasma was stored at −80 °C until required. Plasma levels of ADMA and l-arginine were measured by high-performance liquid chromatography, by precolumn derivatization with ophthalaldehyde, after removal of plasma samples with carboxylic acid solid-phase extraction cartridges (Varian, Harbor City, CA, USA). The coefficients of variation were 5.2% within-assay and 5.5% between-assay; the detection limit of the assay was 0.1 μmol/L.

### 4.5. Forearm Blood Flow Measurements

All studies were begun at 9:00 AM after overnight fasting, with the patients lying supine in a quiet, air-conditioned room (22 °C to 24 °C). Subjects were instructed to continue their regular diet, but caffeine, alcohol and smoking were stopped at least 24 h before the study. For measurement of the vasodilatory response to acetylcholine (ACh), we adopted the protocol by Panza *et al*. [41]. Details and standardization of the technique in our laboratory have already been described in previous publications [9,13,29]. In brief: a 20-gauge polyethylene catheter (Vasculon 2) was inserted into the brachial artery of the nondominant arm under local anesthesia and sterile conditions for evaluation of BP and for drugs infusion. Patients rested 30 min after artery cannulation to reach a stable baseline before data collection and drug infusion. Forearm blood flow (FBF) and BP were measured during intra-arterial infusion of saline, ACh, and sodium nitroprusside (SNP) at increasing doses. Forearm vascular resistance (VR), expressed in arbitrary units (U), was calculated by dividing mean BP at each dose point by FBF. Endothelium-dependent and -independent vasodilations were assessed by a dose-response curve to intra-arterial ACh (7.5, 15 and 30 μg mL^−1^ min^−1^, each for 5 min) and SNP infusions (0.8, 1.6 and 3.2 μg mL^−1^ min^−1^, each for 5 min), respectively. The sequence of administration of ACh and SNP was randomized to avoid any bias related to the order of drug infusion. The drug infusion rate, adjusted for forearm volume of each subject, was 1 mL min^−1^. For the present study, each patient’s FBF maximal response to ACh or SNP was considered for statistical analysis.

### 4.6. Statistical Analysis

Differences for clinical and biological data were compared by using unpaired Student *t-*test and chi-square test. The vasodilatory responses to ACh and SNP were compared by analysis of variance for repeated measurements and, when analysis was significant, the Tukey test was applied. Simple linear regression analysis was performed to assess the relationship between the peak percent increase in FBF in response to ACh infusion, and different covariates (ADMA, HOMA index hs-CRP, l-arginine, systolic and diastolic BP, e-GFR, waist circumference, BMI, HDL-cholesterol, triglyceride, age). In the analysis, we added only HOMA index to avoid a possible colinearity with fasting glucose or insulin. Subsequently, variables reaching statistical significance and gender, as dichotomic values, were inserted in a stepwise multivariate linear regression model to determine the independent predictors of peak percent increase in FBF. The model retained just four variables as statistically significant and was therefore adequately powered (>10 subjects per covariate) to test the hypothesis. Parametric data are reported as mean ± SD. Differences were assumed to be significant at *p* < 0.05. All comparisons were performed using the statistical package SPSS 16.0 for Windows (SPSS, Inc.: Chicago, IL, USA).

## 5. Conclusions

Our results have clinical relevance for understanding the pathogenetic process underlying the development of diabetic disease and its complications. Defining the pathophysiological role of ADMA could lead to therapeutic advancement in reversing endothelial dysfunction and, more importantly, may allow the development of new strategies for the prevention of diabetes and its vascular complications.

This study has some limitations. At first, this is a cross-sectional study thus no causal relationship may be affirmed. Moreover, we have not considered other possible genetic and not genetic factors affecting endothelial function. Finally, the sample size is another possible limitation, but the method used to evaluate endothelial function, also if represents the gold standard, is invasive thus it cannot be easily applicable in a large study population.

## Figures and Tables

**Table 1:** Demographic, humoral and hemodynamic characteristics of the study population stratified by normal or newly diagnosed diabetic status.

	Nondiabetics (*n* = 30)	Newly Diagnosed Type 2 Diabetic (*n* = 45)	*p*
Gender (males/females)	17/13	24/21	0.962 *
Age (years)	45.1 ± 10.6	44.6 ± 7.1	0.807
Body mass index (kg/m^2^)	27.3 ± 1.8	27.8 ± 2.9	0.403
Waist circumference (cm)	92.9 ± 7.4	93.9 ± 3.9	0.448
Systolic BP (mmHg)	125.8 ± 9.3	127.1 ± 8.9	0.545
Diastolic BP (mmHg)	77.7 ± 7.3	79.1 ± 8.3	0.456
Fasting glucose (mg/dL)	90.4 ± 8.6	114.1 ± 15.6	<0.0001
Fasting insulin (μU/mL)	8.6 ± 2.8	15.1 ± 5.4	<0.0001
HOMA	1.9 ± 0.6	5.6 ± 2.1	<0.0001
hs-CRP (mg/L)	1.4 ± 0.8	4.5 ± 1.9	<0.0001
Total cholesterol (mg/dL)	199.7 ± 22.1	194.2 ± 31.7	0.412
LDL cholesterol (mg/dL)	118.8 ± 23.9	123.7 ± 32.3	0.480
HDL cholesterol (mg/dL)	50.3 ± 10.5	42.8 ± 8.9	0.001
Triglyceride (mg/dL)	100.3 ± 41.4	138.6 ± 62.6	0.004
basal FBF (m 100 mL^−1^ of tissue min^−1^)	3.2 ± 0.8	3.1 ± 0.3	0.447
l-arginine (μmol/L)	28.5 ± 7.2	49.8 ± 16.8	<0.0001
ADMA (μmol/L)	0.5 ± 0.2	0.7 ± 0.2	<0.0001
e-GFR (mL/min/1.73m^2^)	99.6 ± 8.4	104.3 ± 15.8	0.140

* χ^2^ test;

BP = blood pressure; HOMA = homeostasis model assessment; CRP = C reactive protein; FBF = Forearm blood flow; ADMA = asymmetric dimethylarginine; e-GFR = estimated glomerular filtration rate.

**Table 2:** Correlational analysis between FBF and different covariates in newly diagnosed type 2 diabetic patients.

	FBF *R/p*
ADMA (μmol/L)	−0.524/<0.0001
HOMA	−0.428/0.002
hs-CRP (mg/L)	−0.416/0.002
l-arginine (μmol/L)	−0.261/0.042
Systolic BP (mmHg)	−0.190/0.105
e-GFR (mL/min/1.73 m^2^)	0.183/0.114
Waist circumference (cm)	−0.156/0.153
Total cholesterol (mmol/L)	0.155/0.154
Body mass index (kg/m^2^)	−0.143/0.175
HDL cholesterol (mmol/L)	0.117/0.221
Triglycerides (mmol/L)	−0.063/0.340
Age (yrs)	0.044/0.387
Diastolic BP (mmHg)	0.024/0.437

FBF = forearm blood flow; HOMA = homeostasis model assessment; hs-CRP =high sensitivity C reactive protein; ADMA = asymmetric dimethylarginine; e-GFR = estimated glomerular filtration rate.

**Table 3:** Independent predictors of forearm blood flow in newly Diagnosed type 2 diabetic patients.

	Partial *R^2^*	Total *R^2^*	*p*
ADMA, μmol/L	27.5	27.5	<0.0001
HOMA	7.0	34.5	0.040

ADMA = asymmetric dimethylarginine; HOMA = homeostasis model assessment.
